# Comparative Study of Crucial Properties of Packaging Based on Polylactide and Selected Essential Oils

**DOI:** 10.3390/foods14020204

**Published:** 2025-01-10

**Authors:** Ewa Olewnik-Kruszkowska, Astha Vishwakarma, Magdalena Wrona, Anis Bertella, Anna Rudawska, Magdalena Gierszewska, Beata Schmidt

**Affiliations:** 1 Chair of Physical Chemistry and Physicochemistry of Polymers, Faculty of Chemistry, Nicolaus Copernicus University in Toruń, Gagarina 7 Street, 87-100 Toruń, Poland; mgd@umk.pl; 2 Institut de Chimie et des Matériaux Paris-Est (ICMPE), Centre National de la Recherche Scientifique (CNRS), Universite Paris-Est Creteil, UMR 7182, 2 Rue Henri Dunant, 94320 Thiais, France; astha.vishwakarma@cnrs.fr; 3 Forschungszentrum Jülich GmbH, Institute of Bio- and Geosciences 2, 52428 Jülich, Germany; m.wrona@fz-juelich.de; 4 Department of Molecular and Cellular Biology, Faculty of Life and Nature Sciences, Abbes Laghrour University Khenchela, BP 1252 Road of Batna, Khenchela 40004, Algeria; anis.bertella@univ-khenchela.dz; 5 Faculty of Mechanical Engineering, Lublin University of Technology, Nadbystrzycka 36 St., 20-618 Lublin, Poland; a.rudawska@pollub.pl; 6 Department of Chemical Organic Technology and Polymeric Materials, Faculty of Chemical Technology and Engineering, West Pomeranian University of Technology, Pułaskiego 10, 70-322 Szczecin, Poland; beata.schmidt@zut.edu.pl

**Keywords:** polylactide, packaging materials, clove oil, tea tree oil, grapefruit oil, rosemary oil

## Abstract

In order to establish the differences in packaging containing various essential oils, polylactide (PLA)-based polymeric films incorporating poly(ethylene glycol) (PEG), clove (C), grapefruit (G), rosemary (R), and tea tree (T) essential oils were obtained and subsequently analyzed. In addition to examining structure and morphology, the polymer films underwent analyses that are particularly important with regard to contact with food. Mechanical and antioxidant properties, water vapor transmission rate (WVTR), and analysis of barrier properties against ultraviolet (UV) radiation, as well as the migration of ingredients into food simulants such as 10% *v*/*v* solutions of ethanol, 3% *w*/*v* acetic acid solution, and isooctane, were among the critical studies conducted. A comparison of the properties of the obtained materials allowed us to establish that the incorporation of essential oils significantly increases elongation at break and enhances UV barrier properties. In the case of materials containing clove oil and tea tree oil, a reduction in WVTR of about 1 g/m^2^/h was observed. The migration of the ingredients present in the films filled with clove oil, grapefruit oil, and tea tree oil into the acetic acid solution did not exceed 10 mg/kg, which is an acceptable value according to the European Union restrictions. Taking into account all of the studied properties, it should be stressed that the most promising packaging material is the film filled with clove oil.

## 1. Introduction

Packaging not only shields products from spoilage or damage, but also serves several additional purposes. It can also promote the product, facilitate its identification, and enable its transportation, storage, and use. Moreover, modern packaging is expected to go beyond these basic, passive roles and perform targeted functions, particularly in preserving food quality. This type of packaging is referred to as active packaging. In most cases, it contains substances that modify the internal package atmosphere or interact directly with the packaged goods. Essential oils are a notable example of such active components. These natural plant-derived substances are obtained mainly through steam distillation or fruit pressing. It should be noted that essential oils have a range of health and care benefits, which is why they are used in various industries [1,2]. However, despite their numerous advantages, the use of essential oils in packaging presents certain challenges. Their volatility, for example, can complicate the controlled release of active substances, hinder interactions with other packaging components, and cause potential changes in mechanical properties. Moreover, compatibility issues with specific polymers can lead to phase separation or uneven distribution [2,3,4,5,6,7,8,9,10]. It is also necessary to comply with regulations regarding food contact safety. For this reason, various methods are used to incorporate essential oils into the polymer matrix.

The properties of polymer materials incorporating essential oils have been described in many studies [8,11,12,13,14,15,16,17,18,19]. Currently, essential oils are introduced into the polymer matrix in various forms. The most common method is direct incorporation into the polymer matrix. This is the fastest and most cost-effective method, but it may have limitations, such as the evaporation of oil components or their overly rapid release into food and the surrounding environment. An example of the formation of such films was presented in the work of Ejaz et al. [20], where clove essential oil was introduced into gelatin. This approach resulted in an improvement in material elasticity and oxygen barrier properties, as well as an extended shelf life of shrimp consumables. In the work of Wozniak et al. [21], chitosan-based films infused with essential oil components such as carvacrol, eugenol, and isoeugenol were presented. In the case of these materials, satisfactory mechanical and antioxidant properties were also achieved, and interactions between chitosan and additional components were identified. In various studies [14,15,16], antibacterial polylactide-based packaging materials have also been formed by incorporating cinnamon, bergamot, lemongrass, clove, tea tree, and oregano oils. The obtained results allow us to establish that essential oils can play a role as plasticizers, as well as leading to the formation of antibacterial packaging.

Recently, attempts have also been made to incorporate essential oils into packaging materials in the form of various emulsions. The effect of clove essential oil nanoemulsion on the physicochemical and antioxidant properties of a chitosan film was described by Rui et al. [13]. The emulsifying properties of clove essential oil in obtaining Pickering emulsions were described by Li et al. [22]. It has been proved that the obtained emulsion can remain stable for as long as 28 days, and that its stability strongly depends on pH. Another method of introducing essential oils into a polymer matrix is to encapsulate and incorporate them into polymeric films [23,24]. Rusková et al. [25] have attempted to encapsulate essential oils, such as lemongrass and oregano, and incorporate them into PLA and poly(3-hydroxybutyrate) (PHB) packaging. Similarly, in the work of Ferreira et al. [26], chitosan-based nanocapsules filled with clove oil were introduced into a poly(butylene adipate-co-terephthalate) matrix. The obtained results confirmed that the applied method allowed the formation of promising antibacterial materials that can be used as active packaging. Another approach to obtaining active films filled with essential oils is the formation of an edible coating [11,27,28,29]. It has been established that edible coatings can be characterized by improved stability and the ability to release active compounds.

In this study, a unique method for the formulation of PLA films modified with PEG and selected essential oils—clove, grapefruit, rosemary, and tea tree—was devised and studied. It is important to emphasize that the oils mentioned above were selected after reviewing the available literature [30,31,32,33,34,35,36,37,38], which indicates that these oils exhibit anti-inflammatory, antioxidant, antibacterial, and antifungal properties. Moreover, selected oils differ in their hydrophilic properties, which can significantly influence the properties of the obtained films.

In the case of the above-mentioned materials, structural, thermal, and mechanical properties were primarily studied. In the present work, a comparative study of the crucial properties of packaging based on polylactide and particular essential oils was conducted. Their global migration into food simulants, UV and water vapor barriers, and antioxidant and mechanical properties—which are critical for food storage—were taken into consideration. Moreover, their structure, morphology, and topography, which are responsible for the behavior of the obtained materials, were established.

## 2. Materials and Methods

### 2.1. Materials

Polylactide in the form of pellets type 2002D, with an average molecular weight of 155,500 Da, was provided by Nature Works^®^ (Minnetonka, MN, USA). All the essential oils (clove oil, grapefruit oil, rosemary oil, tea tree oil) and poly(ethylene glycol) with Mw = 1500 were supplied by Sigma-Aldrich (Steinheim, Germany). Chloroform, methanol, ethanol, acetic acid, isooctane, and calcium chloride were purchased from Avantor Performance Materials Poland S.A. (Gliwice, Poland).

### 2.2. Formation of Polylactide-Based Films

The polylactide-based materials containing essential oils were prepared according to the following procedure. The control film was obtained using a solution consisting of polylactide (3% *m*/*v*) and poly(ethylene glycol) (5% *w*/*w*, PLA) dissolved in chloroform. In the next stage, essential oil (10% *v*/*w*, PLA) was added to 50 mL of the PLA–PEG solution. The mixture was then placed in Petri dishes and left at ambient temperature for 48 h to allow the solvent to evaporate. The resulting formulations were cast onto clean glass plates and left at ambient temperature for another 48 h for solvent evaporation. The following designations were used to identify the individual samples: L—polylactide, P—poly(ethylene glycol), C—clove oil, G—grapefruit oil, R—rosemary oil, and T—tea tree oil. The incorporation of essential oils into the PLA–PEG system did not significantly affect the thickness of the formed films, which ranged from 0.0806 mm for the LP sample to 0.0840 mm for the LPC and LPG samples. The thickness of the obtained films was measured using an Absolute Digimatic Indicator (Sylvac S229, Swiss, Yverdon, Switzerland).

### 2.3. Methods of Analysis

#### 2.3.1. Structure Measurements

The changes in the structure of the obtained materials, resulting from the introduction of essential oils, were studied using a Nicolet iS10 (Thermo Fisher Scientific, Waltham, MA, USA). During the recording of the spectra, 64 scans were applied. A wavenumber range of 600–4000 cm^−1^ and a resolution of 4 cm^−1^ were adopted. The obtained data were analyzed by means of the OMNIC 7.0 software (Thermo Fisher Scientific, Waltham, MA, USA).

#### 2.3.2. Surface and Morphology Measurements

The scanning electron microscope Quanta 3D FEG (FEI Company, Hillsboro, OR, USA) was used in order to evaluate changes in the morphology of the formed films caused by the addition of essential oils. Moreover, atomic force microscopy was applied to observe changes in the topography of the formed films after the addition of essential oils. For this purpose, the atomic force microscope with a scanning SPM probe of the NanoScope MultiMode type (Veeco Metrology, Inc., Santa Barbara, CA, USA) was applied. To observe the roughness and evaluate the root mean square (R_q_) and arithmetical mean deviation (R_a_) of the assessed profile, Nanoscope software (Veeco Metrology, Inc., Santa Barbara, CA, USA) was applied.

#### 2.3.3. Thermal Properties

Differential scanning calorimetry was applied to establish the thermal properties of the studied materials, both with and without the addition of essential oils. The following conditions were used: nitrogen atmosphere, a heating rate of 10 °C/min, and temperatures ranging between 25 and 200 °C. During the measurements, the thermoanalyzer manufactured by Polymer Laboratories (Epsom, UK) was used.

#### 2.3.4. Mechanical Properties

Analysis of the effect of the essential oils on the mechanical properties (such as Young’s modulus (E), elongation at break (ε), and tensile stress (σ_m_)) of the PLA-based materials was performed using the EZ-SX machine (Shimadzu, Kyoto, Japan). The crosshead speed was 10 mm·min^−1^ with an applied 100 N force. The samples for testing mechanical properties were cut in the shape of a paddle. The width of the analyzed sample was 5 mm. For each sample, the analysis was performed on five paddles.

#### 2.3.5. Assessment of Antioxidative Properties

The antioxidative potential of the PLA-based materials filled with different essential oils was established using the DPPH method. The analyses were performed according to the methodology described in the work of Ouahioune et al. [39]. A total of 0.6 g of every film was introduced into 6 mL of methanol and shaken for 20 min. Five concentrations of the obtained extracts were studied for every polymeric film. The concentration of DPPH was 30 µg/g. After 30 min of incubation in the dark, the absorbance of the stored solutions was studied at 515 nm using a Halo DB-20 spectrophotometer (Dynamica Scientific Ltd., Newport Pagnell, UK). The plotted graphs of scavenging activity against the volume of the extracts allowed us to estimate the volume of extract providing 50% inhibition (IC_50_). All the absorbance measurements were performed in triplicate.

#### 2.3.6. Water Vapor Transmission Rate

The influence of different essential oils on the water vapor transmission rate of PLA-based films was established according to the methodology presented in our previous work [40]. For this reason, the changes in the calcium chloride weight were measured every 24 h for seven days. During the analysis, the relative humidity was 75%, while the temperature of the ambient environment during storage was 30 °C. A triplicate analysis was conducted in the case of every sample. In the aim of evaluating the effect of clove oil, grapefruit oil, rosemary oil, and tea tree oil on WVTR, the following equation was used (1):

(1) $$\mathrm{WVTR}=\frac{rate\;of\;moisture\;absorption\;by\;dessicant}{surface\;area\;of\;specimen}\left[\frac{g}{m^{2}\times h}\right]$$

#### 2.3.7. Global Migration Test

Migration tests belong to the basic set of tests that materials and products intended for contact with food are subject to. For this reason, the indications of the European Commission Regulation (EU) 2016/1416 [41] on appropriate food simulants and conditions were applied. In the aim of establishing global migration, three food simulants were used: 10% *v*/*v* solutions of ethanol (Food simulant A), 3% *w*/*v* acetic acid solution (Food simulant B), and isooctane (Food simulant D). During the analysis, the ratio of film surface to solution mass was 6 dm^2^/1 kg. A triplicate analysis was conducted for each sample. The global migration was calculated by the changes in the weight of polymeric films before and after storage in food simulants for 10 days at 40 °C.

#### 2.3.8. UV Protection

In the aim of establishing protection against UV radiation, the optical properties of all of the studied materials were determined. Spectra were obtained in the range of 200–800 nm by means of a Halo DB-20 spectrophotometer (Dynamica Scientific Ltd., Newport Pagnell, UK). A triplicate analysis was conducted for each sample. UV-blocking activity was studied in the UVA and UVB ranges, and for this reason, the following Equations (2) and (3) were applied:

(2) $$UV-A_{blocking}=100-T_{UV-A}$$

(3) $$UV-B_{blocking}=100-T_{UVB}$$

where *T_UVA_* is the average transmittance value in the UVA region (from 400 nm to 320 nm), and *T_UVB_* is the average transmittance value in the UVB region (from 320 to 290 nm) [40].

#### 2.3.9. Statistics

A Student’s *t*-test was used to assess whether there were significant (*p* ≤ 0.05) differences between the films containing different essential oils. The null hypothesis was that there would be no difference between the samples. If the calculated *t*-value exceeded the critical t-value from the statistical table, the samples were considered significantly different, and the null hypothesis was rejected.

## 3. Results and Discussion

### 3.1. SEM and AFM Analysis

**Figure 1:**
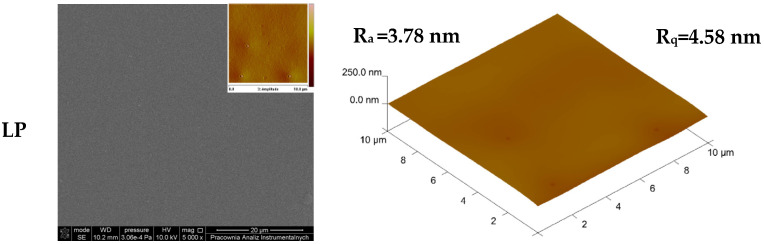

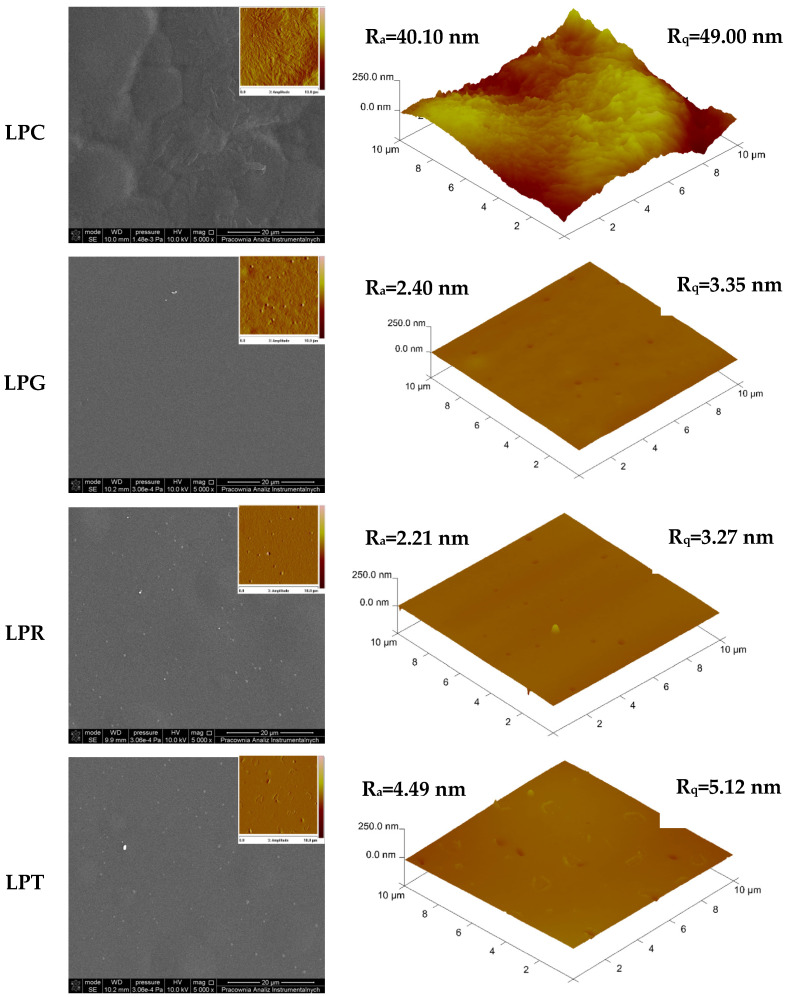
SEM and AFM images of the formed PLA-based materials (LP is a PLA–PEG system filled with clove oil—LPC, grapefruit oil—LPG, rosemary oil—LPR, and tea tree oil—LPT); magnification 5000×.

It is well known that additives can significantly influence the morphology, microstructure, and topography of the obtained materials [27,42,43]. For this reason, scanning electron microscopy and atomic force microscopy were applied to obtain SEM and AFM images, as well as R_a_ and R_q_ values pertaining to the studied materials, as depicted in Figure 1.

**Figure 2:**
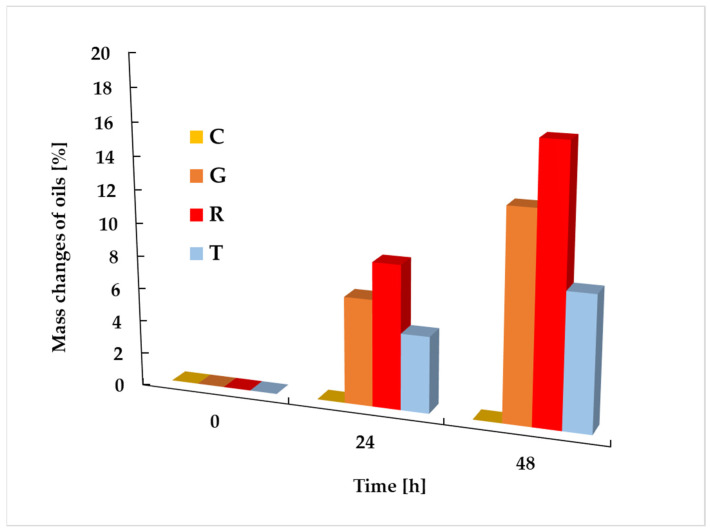
Mass changes of used essential oils (C—clove oil, G—grapefruit oil, R—rosemary oil, T—tea tree oil) during two days of storage in ambient conditions.

The surface of the control film, consisting of PLA and PEG, was smooth and free of cracks and irregularities, as indicated by the low values of the R_a_ and R_q_ parameters. In contrast, the morphology and topography of the films containing essential oils differed significantly depending on the type of additive used. The samples containing grapefruit oil, rosemary oil, and tea tree oil were covered with small holes. A similar effect has been observed in other studies on polymer films with the addition of these essential oils [44,45]. It can be assumed that this is caused by the evaporation not only of the solvent, but also of the volatile components of the essential oils introduced into the PLA–PEG matrix. To confirm this assumption, an analysis of the mass loss of the oils was conducted in the same conditions as the ones in which the polymeric films were formed. The change in the mass of the oils was recorded after 24 and 48 h. The obtained results of the change in mass of the essential oils used are depicted in Figure 2.

The studies revealed that after both 24 and 48 h during the formation of the polymer films, the greatest mass loss was observed in rosemary oil, followed by grapefruit oil and tea tree oil. In the same order, the values of the R_a_ and R_q_ parameters increased, which may confirm that the evaporation of components could lead to the formation of holes and may also affect the topography of the materials. To explain the changes in morphology and topography following the introduction of various oils into the PLA–PEG system, the hydrophilicity and hydrophobicity of the material components were also considered. Given that oils are complex multicomponent systems, interactions between polylactide and only the primary oil component, i.e., the one present in the highest quantity, were analyzed. Based on the published data [2,8,9,46,47,48,49,50], the main components of the utilized oils were ranked from the most hydrophobic to the most hydrophilic. Among the oils studied, the most hydrophobic component turned out to be limonene, which is the primary component of grapefruit oil. Its hydrophobicity is attributed to the absence of polar groups, making it practically insoluble in water. Next is eucalyptol (1,8-cineole), which is partially hydrophobic and the main component of rosemary oil. Notably, it contains an ether group, which increases its polarity compared to limonene but still classifies it as moderately hydrophobic. Terpinen-4-ol, the main component of tea tree oil, also exhibits partial hydrophobicity. The presence of a hydroxyl group gives it higher polarity compared to eucalyptol, making it more hydrophilic. The most hydrophilic component among the main constituents of the oils studied is eugenol, the primary component of clove oil. Eugenol contains both hydroxyl and ether groups, which significantly enhance its polarity and water solubility compared to the other compounds. Considering that polylactide is a hydrophobic polymer, interactions between this polymer and the main oil components can also influence the morphology and topography of the resulting films. Materials containing grapefruit and rosemary oils exhibited the smoothest surfaces. The introduction of tea tree oil into the PLA–PEG system slightly increased the material’s roughness. In contrast, the film incorporating clove oil, whose composition included 70–90% eugenol, had the roughest and most wrinkled surface. Despite the high hydrophilicity of clove oil’s main component, no phase separation was observed in the obtained LPC sample.

### 3.2. Influence of Essential Oils on Structure

**Figure 3:**
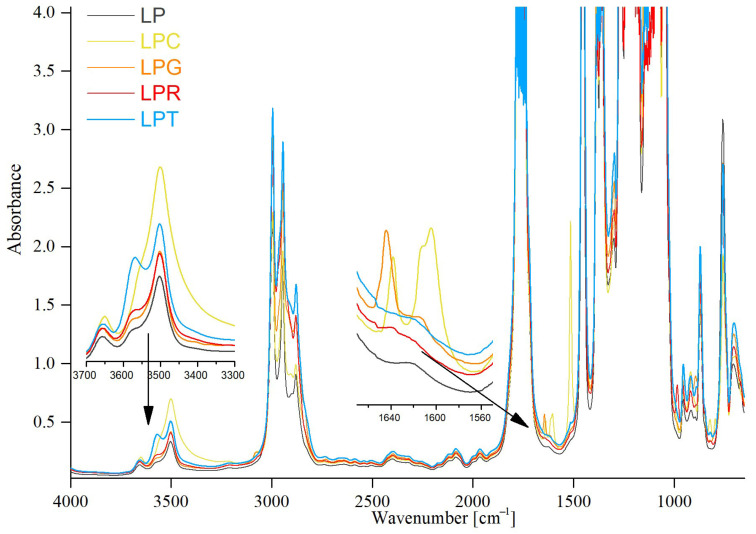
Spectra of analyzed films without and with addition of essential oils (LP is a PLA–PEG system filled with clove oil—LPC, grapefruit oil—LPG, rosemary oil—LPR, and tea tree oil—LPT).

The FTIR analysis allowed for the determination of the effect of the essential oils on structural changes in the obtained PLA-based materials. The spectra of the studied materials can be observed in Figure 3. In our previous works [43,51], the structure of PLA film, as well as that of PLA with the addition of poly(ethylene glycol), was carefully described. However, the addition of all essential oils caused changes in the number and range of the individual bands. According to the available literature, the most significant changes in band intensity and the appearance of new bands after the incorporation of the indicated oils are described.

The most notable changes in the spectrum were observed after the introduction of clove oil into the polymeric matrix. It is well known that clove oil mainly consists of eugenol‚ caryophyllene, R-humulene, and eugenol acetate. The listed ingredients of clove oil constitute about 99.5% of its total composition, while the remaining ingredients are only 0.5% [52]. The most significant changes occurred in the area at around 1637 cm^−1^ and around 1610 cm^−1^, and are in line with the FTIR spectrum of pure eugenol [53,54,55,56]. The new bands at 1637 cm^−1^ and 1610 cm^−1^ belong to the aromatic C=C bond. Moreover, an increase in the band’s intensity at 3500 cm^−1^, representative of the –OH vibration in phenol groups, can be seen. Eugenol also shows changes at 3100 cm^−1^ and 2850–3000 cm^−1^. The indicated bands can be attributed to aromatic C–H stretching and symmetric and asymmetric stretching of methyl and methylene groups, respectively.

Recent studies [57,58,59] indicate that limonene terpenoids belong to the main compounds of grapefruit oil. Bands characteristic of the mentioned ingredients can be observed at 1644 cm^−1^ as well as at 1453 cm^−1^. Moreover, it should be stressed that there are no changes in the region corresponding to −OH groups. In the case of rosemary oil, the expected characteristic bands at ~2900 cm^−1^ (C-H stretch), ~1700 cm^−1^ (C=O stretch), ~3400 cm^−1^ (−OH stretch), and ~1100 cm^−1^ (C-O stretch), indicative of the terpenoid components of the oil, are almost completely overlapped with the groups present in PLA and PEG [60,61,62,63]. For this reason, it can be concluded that the incorporation of rosemary oil in the PLA–PEG system does not change significantly the spectrum of PLA–PEG film.

The FTIR spectrum of tea tree essential oil was studied in detail by Yadav et al. [38]. Based on FTIR analysis, the functional groups and compound classes of tea tree oil were established. According to Yadav [43], terpinen-4-ol and 1,8-cineole constitute the main compounds present in this oil. The same observation was made by Borotová et al. [64].

In summary, the composition of the introduced essential oils can vary significantly depending on the conditions and the environment in which the plants that are the source of the oils are cultivated. However, it should be emphasized that the main components of the essential oils remain consistent, regardless of the crop.

It is important to point out that the incorporation of clove oil, grapefruit oil, and rosemary oil, as well as tea tree oil, into the PLA–PEG matrix does not involve the formation of new bonds, but only the appearance of bands characteristic of the components of the oils used.

### 3.3. Determination of Thermal Properties of PLA-Based Materials

**Figure 4:**
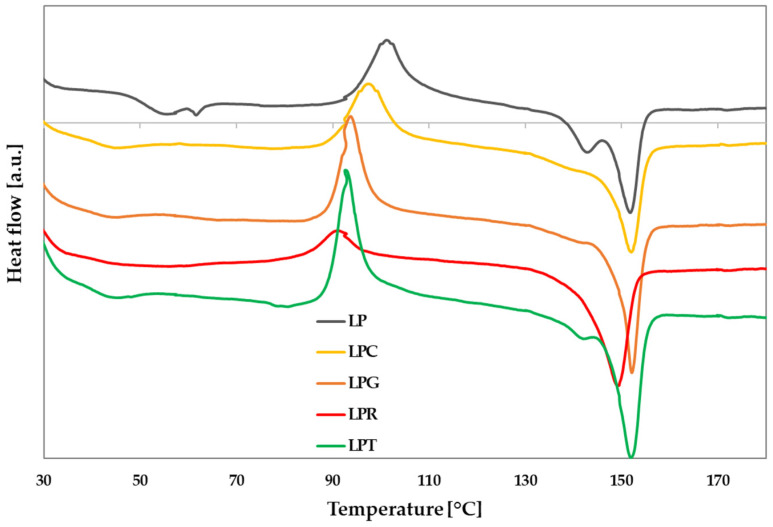
DSC curves of the analyzed polymeric materials (LP is a PLA–PEG system filled with clove oil—LPC, grapefruit oil—LPG, rosemary oil—LPR, and tea tree oil—LPT).

The incorporation of different kinds of additives into the polymeric matrix, in most cases, changes the thermal properties of the obtained polymeric films. In Figure 4, the thermograms of studied PLA–PEG systems with and without the addition of different essential oils are depicted. Additionally, the values of degree of crystallization (X), cold crystallization enthalpy (ΔH_c_), and melting enthalpy (ΔH_m_), as well as the temperatures of glass transition (T_g_), cold crystallization (T_c_), and melting (T_m_), were determined, and are shown in Table 1. Based on the presented data, it can be observed that the addition of the same amount of different essential oils can result in significantly different effects on the thermal parameters of the obtained materials. In the case of all materials, after introducing the essential oil into the polymer matrix, decreases in parameters such as crystallization temperature, crystallization enthalpy, melting temperature, and melting enthalpy were observed, indicating a plasticizing effect of the applied essential oils [15]. Moreover, the addition of rosemary oil, grapefruit oil, and tea tree oil led to a significant decrease in glass transition temperature, due to an increase in the mobility of the polymer chains. On the other hand, no glass transition temperature was recorded within the studied temperature range after the introduction of clove oil. In the work of Qin et al. [15], in which various essential oils such as bergamot, lemongrass, rosemary, and clove oils were incorporated into the polymer matrix, it was also observed that films containing clove oil exhibited the lowest glass transition temperature.

Similarly, varied values were obtained for the melting enthalpy of the systems containing essential oils. Interestingly, these values remained high despite the addition of the oils.

This effect, similary to the morphology of the obtained films, may depend on the affinity between the components of particular samples. As mentioned earlier, the hydrophobic and hydrophilic characteristics associated with the structure of the main components of the oils used were analyzed, taking into account the available literature [2,8,9,46,47,48,49,50]. These components were then ranked with regard to hydrophilicity and hydrophobicity. The analysis showed that eugenol is the most hydrophilic constituent and exhibits the lowest affinity to polylactide, leading to irregularities on the surface of the obtained LPC film. This may reduce the interactions between the PLA chain and eugenol, which in turn results in the absence of a glass transition temperature for the LPC sample and the lowest degree of crystallinity among all of the studied systems. Moreover, for this material, the melting enthalpy reached the lowest value among the systems containing essential oils. Similar observations regarding materials with the addition of clove oil were made by Ahmed et al. [65]. It should also be noted that only when introducing grapefruit oil into the PLA–PEG system was a distinct presence of two melting temperatures observed, indicating the presence of different crystalline forms of polylactide [51]. The same tendency, involving a decrease in the values of all the discussed parameters, was observed in a study by Cerdá-Gandia et al. [66].

### 3.4. Changes in Mechanical Properties

The mechanical properties of polymer materials are strongly dependent on the type of polymers and introduced additives. From a utility perspective, the analysis of mechanical properties is one of the key parameters to be considered when selecting packaging for a particular product.

**Figure 5:**
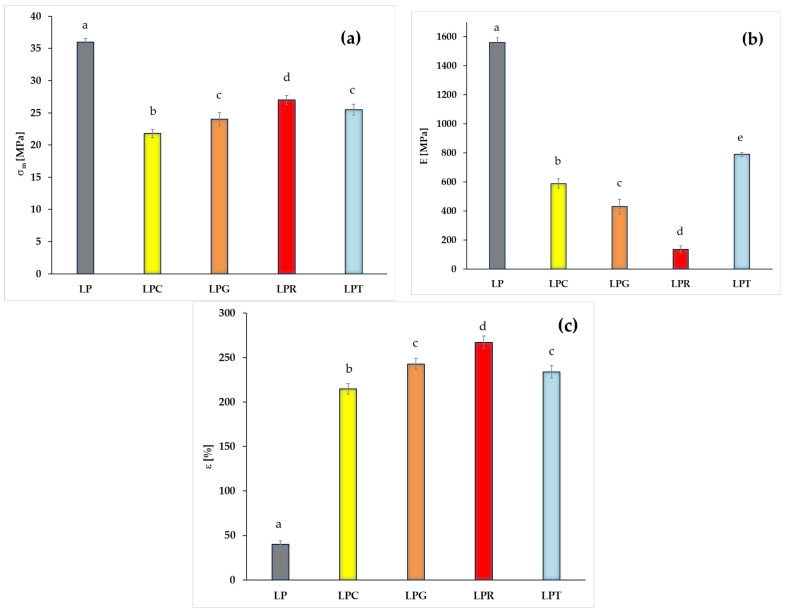
Changes in mechanical properties of PLA-based films with and without different essential oils (LP is a PLA–PEG system filled with clove oil—LPC, grapefruit oil—LPG, rosemary oil—LPR, and tea tree oil—LPT): (**a**) Young’s Modulus (E), (**b**) tensile strength (σ_m_), and (**c**) elongation at break (ε) (different letters (a–e) indicate significative (*p* ≤ 0.05) differences between samples).

Therefore, in the following section of the study, the impacts of different essential oils, namely clove oil, grapefruit oil, rosemary oil, and tea tree oil, on parameters including tensile strength (σ_m_), Young’s Modulus (E), and elongation at break (ε) (Figure 5) were discussed.

Considering the fact that essential oils are often used as a plasticizer [17], a significant decrease in the Young’s modulus value was observed for all the films containing essential oils compared to the control sample. However, the reduction in this parameter occurred to varying degrees depending on the essential oil used. The lowest value of Young’s modulus, 136 MPa, was observed in the case of the material filled with rosemary oil. In contrast, for the films with the addition of clove oil and tea tree oil, the Young’s modulus values were 589 MPa and 790 MPa, respectively, several times higher than for the sample filled with rosemary oil. It is worth noting that the Young’s modulus values for the LPC and LPT samples were the highest, despite these samples having the lowest crystallinity values. A similar decrease in the tensile strength values was observed for the materials with the addition of essential oils compared to the control sample. However, the differences in the values of this parameter between the films filled with different essential oils were not as significant as those observed for Young’s modulus. Similar trends were described in the work of Sanchez-Gonzalez et al. [44], in which edible films based on hydroxypropylmethylcellulose and tea tree essential oil were studied. Furthermore, a similar effect of adding essential oils to various polymers has been reported by other researchers [15,67].

It should be stressed that after the introduction of the studied essential oils, a significant increase in elongation at break was observed. The highest value of ε was recorded for the sample containing rosemary oil (267%), while the lowest elongation at break values were noted for the LPC (215%) and LPT (234%) samples.

Mixing compounds with differing hydrophilic–hydrophobic characteristics presents a challenge in terms of maintaining particular mechanical properties of the resulting systems. It is important to note that essential oils are complex systems composed of components with varying affinities in relation to the polymer matrix. It should be stressed that the addition of a compound with increased hydrophilicity can lead to the formation of regions with reduced cohesion, which in turn lowers tensile strength [68,69,70,71]. Considering that the main component of clove oil exhibits the highest hydrophilicity among the tested oils, the observations align with the original premise, as the LPC sample is characterized by both the lowest elongation at break and the lowest tensile strength.

The obtained results suggest that, in the case of materials containing essential oils, the parameters that can determine the mechanical properties include not the degree of crystallinity, but likely the type and the evaporation rate of the volatile components of the essential oils.

### 3.5. Examination of WVTR

**Figure 6:**
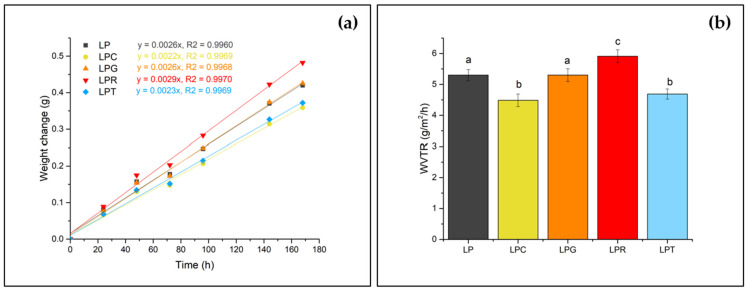
(**a**) Relationship between time and desiccant weight change; (**b**) water vapor transmission rates for the various specimens (different letters (a–c) indicate significant (*p* ≤ 0.05) differences between samples) (LP is a PLA–PEG system filled with clove oil—LPC, grapefruit oil—LPG, rosemary oil—LPR, and tea tree oil—LPT).

Changes in desiccant weight over time demonstrated a strong positive Pearson correlation coefficient (R) across all specimen types (Figure 6a). It can be clearly seen that the incorporation of clove essential oil significantly improved the water vapor resistance of the polylactide-based film, achieving a 24% reduction in WVTR compared to the pristine PLA–PEG sample. Tea tree essential oil also demonstrated a notable reduction (−11%), while grapefruit essential oil maintained the same WVTR as the control. However, the addition of rosemary essential oil led to a 26% increase in WVTR.

These findings are consistent with those observed in films based on polysaccharides and proteins. For instance, Djebbi et al. [72] reported similar enhancements in water vapor resistance with essential oils such as bitter orange, basil, green cardamom, and black pepper incorporated into chitosan films, evaluated over a 35-day period. Another study [73] highlighted a significant reduction in WVTR with the addition of just 1% *Ziziphora clinopodioides* and grape seed extract in chitosan- and gelatin-based films. In terms of water vapor permeability (WVP)—calculated by normalizing the WVTR with the average film thickness and partial pressure difference under steady-state permeation conditions—the binary polysaccharide network of carboxymethylcellulose and chitosan showed a remarkable 41–94% reduction in WVP upon incorporating cinnamon and ginger essential oils [74]. Conversely, previous research [15] on PLA blend films containing 9% bergamot, lemongrass, rosemary, and clove essential oils has reported their increased water vapor permeability compared to pristine PLA films. However, it should be stressed that the films mentioned above were formed without using a plasticizer, which significantly influences the water vapor transmission rate [75,76,77]. According to the available literature [76,77,78], water vapor permeability can largely be determined by the degree of crystallinity. In the case of the studied materials, the films with the addition of clove oil and tea tree oil, despite having the lowest crystallinity values, exhibited the highest resistance to water vapor permeability. Therefore, it can be assumed that the aforementioned general rule does not apply to films containing essential oils. For the tested materials, additives were introduced that not only affect the degree of crystallinity, but also possess hydrophobic properties [48,79]. Thus, incorporating hydrophobic compounds into the polymer matrix may strengthen the material’s resistance to water vapor permeability, regardless of the crystallinity level of the resulting packaging.

### 3.6. Differences in Antioxidative Properties

**Figure 7:**
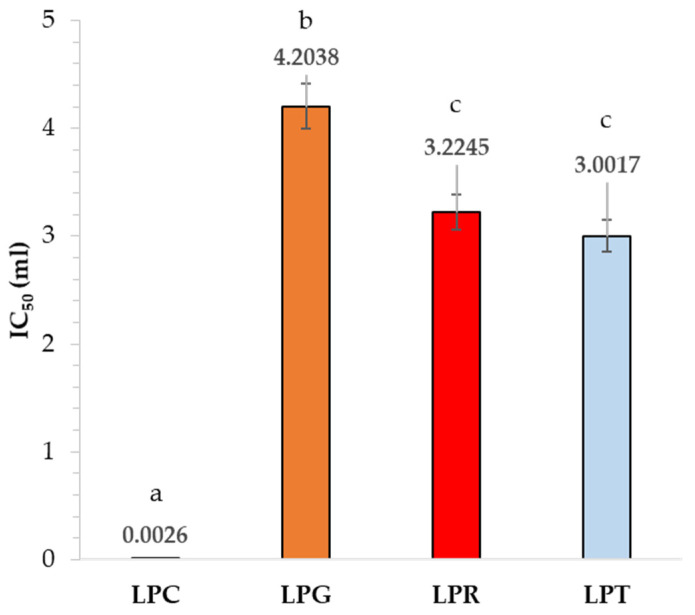
Results of antioxidant activity of developed active films (PLA–PEG system filled with clove oil—LPC, grapefruit oil—LPG, rosemary oil—LPR, and tea tree oil—LPT) (different letters (a–c) indicate significant (*p* ≤ 0.05) differences between samples).

Figure 7 presents the results from the DPPH method, by which the lowest IC50 value was obtained, and the strongest antioxidant activity of the active film could be observed. The strongest antioxidant activity was established in the case of the film containing clove oil, with IC50 = 0.0026 mL, while for the films containing rosemary and tea tree oils, the antioxidant activity was almost the same, with IC50 = 3.22 mL and IC50 = 3.00 mL, respectively.

The main compound of clove essential oil is eugenol. A strong correlation between the antioxidant activity, determined by the DPPH method, of this essential oil and its primary constituents has been discussed in the literature [80]. Research on biodegradable gelatin films with various essential oils incorporated, including clove oil, has demonstrated their enhanced antioxidant activity. One study found that films containing clove essential oil exhibited higher antioxidant activity compared to those with other essential oils, as measured by a DPPH assay [81].

Rosemary essential oil is known for its antioxidant properties, attributed to compounds such as carnosic acid and rosmarinic acid. The IC₅₀ values in DPPH assays discussed in previous studies suggest that it has a moderate antioxidant activity [82]. Since tea tree oil contains terpenes like terpinen-4-ol and α-terpinene, its antioxidant activity is generally considered to be less pronounced than that of clove and rosemary oils [83,84]. Nonetheless, in this study, a slightly enhanced antioxidant activity of the film containing tea tree oil was observed. The interaction between the components of the film and tea tree oil might create a synergistic effect, amplifying the antioxidant activity. Grapefruit essential oil contains compounds like limonene and linalool [85]. Generally, citrus oils tend to have a moderate to low antioxidant capacity compared to oils rich in phenolic compounds [86]. Therefore, the film containing this oil was justifiably expected to have the lowest antioxidant activity.

### 3.7. Evaluation of Global Migration

**Figure 8:**
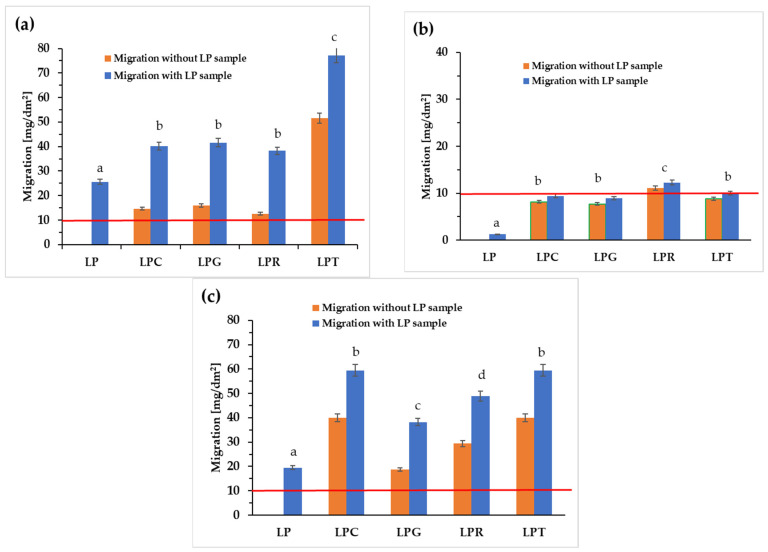
Evaluation of global migration tests in (**a**) ethanol, (**b**) acetic acid, and (**c**) isooctane, (different letters (a–d) indicate significant (*p* ≤ 0.05) differences between samples).

The approval of packaging for contact with food is dependent on compliance with a series of regulations introduced by individual countries as well as by the European Union. One of the most important documents is the European Commission Regulation (EU) 2016/1416 [41], which clearly states that global migration, meaning the sum of the total migration of all the packaging components, into food simulants must not exceed 10 mg/dm^2^ of the applied packaging [5,8,87,88]. It is widely known that depending on the type of food being stored, the packaging may or may not meet the required standards. For this reason, Figure 8 presents the results of global migration for the polylactic acid-based materials both with and without the addition of various essential oils.

Figure 8a shows the migration of components from the film into 10% (*v*/*v*) ethanol, which is a liquid that simulated a hydrophilic environment, and can therefore be considered to mimic fruits that have not been peeled or cut. The migrations are presented in two series: one (blue series) includes the migration of all the components of the tested materials, while the other (orange series) indicates the migration of components from the individual essential oils. Regardless of whether only the migration of the components of the induvial essential oils or the total global migration are considered, it can be observed that for all the materials, the permissible migration limit was significantly exceeded. It is well known that essential oils dissolve in ethanol, so it is not surprising that when the oils came into contact with this medium, their components migrated into the ethanol solution. Given that it was an aqueous ethanol solution, it is also not surprising that components of the base film (most likely PEG and polylactide oligomers) migrated into the applied system.

Figure 8b presents the data obtained during the migration test into an acetic acid solution, which simulates food with a pH below 4.5. In this case, the total global migration of all the components from the tested films also exceeded the acceptable limits, while the migration of components from clove, grapefruit, and tea tree oils was below 10 mg/dm^2^, and the migration of components from rosemary oil was at the threshold of acceptability. This suggests that incorporating these essential oils into a different polymer matrix could lead to the development of packaging that is approved for contact with food with a pH below 4.5. An interesting effect was obtained for global migration in isooctane, which simulates fatty food (Figure 8c), as in this case, the migration values for the samples containing essential oils (except for the LPT sample) reached the highest values, both for the control sample and for the materials containing clove oil, grapefruit oil, and rosemary oil. In summary, the analysis of global migration into the three liquids simulating food showed that only for food with a pH below 4.5 is it possible to use PLA-based packaging containing 10% clove, grapefruit, and tea tree oils. However, it should be noted that essential oils are secondary metabolites of plants. For this reason, they seem to be safer than synthetic preservatives [89]. However, due to the complexity of the ingredients in these oils and the conclusions drawn from toxicological assessments, a thorough evaluation of their safety is essential before using them in food packaging [28,90].

The differences in migration observed for the individual essential oils are undoubtedly related to both their composition and the type of selected food simulant. In the case of multi-component systems such as essential oils, which vary in the type of their components, their hydrophilic–hydrophobic nature, and their concentration, the analysis of the migration results proves to be highly complicated. Significant migration is typically observed when films are exposed to solutions with varying degrees of hydrophobicity, as individual oil components may exhibit differing affinities towards each solution. Moreover, the migration results are strongly dependent on the concentration of particular ingredients within the oils [91,92,93].

Furthermore, it is reasonable to assume that migration is not only determined by the hydrophobic–hydrophilic nature of the components of oils and food simulant liquids. This parameter is also significantly influenced by the content of oils in the polymer matrix, which in turn affects the difference in chemical potential between the components of both phases: solid (polymeric film) and liquid (food simulant) [91,93]. Moreover, the chemical nature of the polymeric matrix can also play an important role (e.g., the type of polymer used, the type and amount of plasticizer), as well as the supermolecular and molecular structure of the tested films. All the above-mentioned parameters affect swelling and the diffusion of the oils from the films to the external media.

### 3.8. UV Barrier Properties

**Figure 9:**
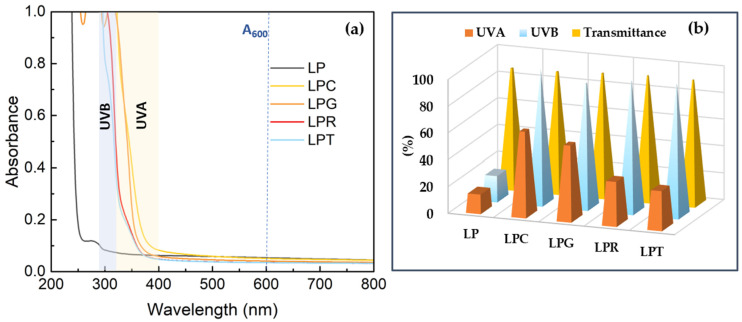
(**a**) UV-visible spectra; (**b**) UV-blocking factors and transmittance at 600 nm of studied materials.

The main factors that cause food spoilage are the presence of microorganisms, elevated temperatures, the presence of oxygen, and light exposure, particularly UV radiation. Therefore, special attention is currently given to packaging that can block UVA (320–400 nm) and UVB (290–320 nm) radiation, which can cause the degradation of food ingredients, negatively affecting their taste, smell, and ultimately their esthetic qualities, as well as, most importantly, eventually leading to terminal product spoilage. For this reason, in order to create packaging with an effective UV barrier, compounds are added during the packaging production process that limit the exposure of the food product to radiation while not significantly increasing the thickness of the polymer film. UV-resistant packaging is particularly beneficial in situations in which the contents are exposed to sunlight, such as in the food or cosmetics industries. For food products like fruit, vegetables, or meat, proper UV protection helps to prevent a loss of nutritional value and changes in color or texture [94,95]. Therefore, UV barrier properties are one of the most crucial and desirable features of packaging intended for food storage. This study investigated the effect of essential oils on the barrier properties and transparency of PLA–PEG-based films. Figure 9a shows the absorption spectra of radiation in the visible and UV ranges for the obtained materials. It should be emphasized that the introduction of essential oils does not affect the permeability of visible radiation, but significantly improves the blocking of UV radiation. In the case of UVB radiation, nearly 100% protection was achieved for all the materials containing essential oils, whereas the PLA–PEG-based film provided only 20% protection against this radiation (Figure 9b). However, in the UVA range, although the tested materials showed an improvement in barrier properties compared to the control sample, this protection was not as effective as in the case of UVB radiation.

The best barrier properties were obtained for the sample containing clove oil. Given that essential oils are rich in phenolic compounds, which significantly improve UV resistance [94,95,96], it is not surprising that they enhance the barrier properties of packaging materials to such a great extent. However, it is worth noting that despite the improvement in UV barrier properties, the obtained films remain largely transparent, as indicated by the high transmittance value at 600 nm.

## 4. Conclusions

Considering that packaging containing essential oils is currently of considerable interest to researchers, this work analyzed polylactide-based materials with the addition of four different essential oils. The effects of the additives on the structure and crucial properties of the food packaging materials were compared. The conducted analyses revealed that although essential oils do not affect the structure of the polylactide-based films, they significantly alter the thermal properties and topography of the formed packaging. All the introduced essential oils (clove oil, grapefruit oil, rosemary oil, and tea tree oil) decreased the glass transition temperature and the degree of crystallinity of the obtained materials. Moreover, the introduction of essential oils into the PLA–PEG matrix significantly improved the flexibility of the obtained films. Adding clove oil and tea tree oil—oils whose components evaporate the least during the film formation process—resulted in increased resistance to water vapor penetration, which is especially important in the case of food storage. In addition, essential oils containing phenolic compounds significantly improved the resistance of the materials to UV radiation, in particular in the UVB range. Unfortunately, global migration tests, conducted by applying food simulants such as acetic acid (3% *w*/*v*), ethanol (10% *v*/*v*), and isooctane, showed that only in the case of fatty foods can clove, rosemary, and grapefruit oils can be used as packaging components. Based on the obtained results, it is evident that the most promising material seems to be the packaging containing clove oil. The film incorporating clove oil demonstrates superior properties, including strong antioxidative potential, improved UV protection, and enhanced water vapor resistance.

In summary, active packaging ensures longer-lasting freshness of food products and directly protects the packaged items from the harmful effects of oxygen, light, and microorganisms. It should be noted that the use of essential oils in packaging intended for food contact is regulated by authorities such as the FDA (Food and Drug Administration) in the United States and the EFSA (European Food Safety Authority) in the European Union. Both the FDA and EFSA approve the use of certain essential oils in packaging; however, their application is strictly conditional upon meeting specific safety requirements, limitations on migration into food, and overall toxicity standards.

Most of the tested essential oils have already been approved by the FDA and EFSA. However, research indicates that their concentration in polymeric materials should be below 10%. Using essential oils in smaller amounts could enable the production of packaging that meets the stringent limits of migration into food simulants. Another promising solution involves the encapsulation of essential oils. Introducing nanocapsules containing essential oils may effectively reduce the rate of essential oil components permeating directly into food.

## Figures and Tables

**Table 1:** DSC data for studied materials (LP is a PLA–PEG system filled with clove oil—LPC, grapefruit oil—LPG, rosemary oil—LPR, and tea tree oil—LPT).

Sample	T_g_ (°C)	T_c_ (°C)	ΔH_c_ (J/g)	T_m_ (°C)	ΔH_m_ (J/g)	X_c_ [%]
LP	51.09	101.11	22.01	142.82/151.79	27.44	26.50
LPC	-	91.16	15.45	149.30	18.6	20.08
LPG	42.68	92.68	19.92	141.22/151.92	23.14	24.98
LPR	39.19	97.33	18.21	152.09	21.18	22.86
LPT	39.03	93.67	16.08	152.09	19.12	20.64

## Data Availability

The original contributions presented in the study are included in the article, further inquiries can be directed to the corresponding author.
